# Antimigration and Anti-Invasion Properties of *Aspergillus aculeatus* Extract, an Endophyte Isolated From *Capsicum annuum* L. on Non–Small-Cell Lung Cancer Cells: *In Vitro* Experiments and *In Silico* Methods

**DOI:** 10.1155/sci5/5676577

**Published:** 2025-08-21

**Authors:** Malatee Tayeh, Imran Sama-ae, Sueptrakool Wisessombat, Wipawadee Sianglum

**Affiliations:** ^1^Department of Medical Technology and Hematology and Transfusion Science Research Center, Walailak University, Nakhon Si Thammarat, Thailand; ^2^Department of Medical Technology and Center of Excellence Research for Melioidosis and Microorganisms, Walailak University, Nakhon Si Thammarat, Thailand; ^3^Division of Biological Science, Faculty of Science, Prince of Songkla University, Songkhla, Thailand

## Abstract

Endophytic fungi are microorganisms that infect living plant tissues internally without producing obvious symptoms of infection, existing in a symbiotic relationship with plants for a portion of their life cycle. Currently, endophytic fungi serve as alternate sources for the production of new bioactive chemicals with great efficacy. This study aimed to examine the antimigration and anti-invasion capabilities of the endophytic fungus *Aspergillus aculeatus* extract, isolated from *Capsicum annuum* L., utilizing *in vitro* and *in silico* methods. This study isolated the endophytic fungus *A. aculeatus* from the leaves of *C. annuum* L. LC–MS analysis revealed fifty-five active components within the extract. Ten compounds exhibited favorable results in the *in silico* assessment. Computational predictions indicate that tajixanthone methanoate (−8.80 kcal/mol) and aspernigerin (−12.95 kcal/mol) exhibited high binding affinity against MMP-2. The *A. aculeatus* extract demonstrated antiproliferative activity with an IC_50_ value of 286.36 ± 122.57 μg/mL. The extract, at noncytotoxic concentrations, reduced the migration and invasion of A549 cells in a dose-dependent manner. Furthermore, *A. aculeatus* extract demonstrated a marked reduction in MMP-2 activity. According to these results, the compounds may serve as antimigration and anti-invasion agents by inhibiting the MMP-2 protein. The results demonstrated that *A. aculeatus* extract derived from *C. annuum* L. inhibited A549 cell migration and invasion via reducing MMP-2 activity. The findings indicated that *A. aculeatus* extract derived from *C. annuum* L. may be utilized for the treatment of lung cancer.

## 1. Introduction

Worldwide, lung cancer is the major cause of cancer-related death [1]. It is divided into two types: small-cell lung cancer (SCLC) and non–small-cell lung cancer (NSCLC). NSCLC is the most common type of lung cancer, accounting for over 80% of all cases. It is divided into adenocarcinoma, squamous cell carcinoma, and large-cell carcinoma [2, 3]. Metastasized lung cancer is difficult to treat because it is highly resistant to radiation and conventional chemotherapeutic agents. It is a main cause of cancer-related death in lung cancer patients. NSCLC has a high tendency for metastasis, with a 5-year survival rate of 10%–15% in patients with metastatic lung cancer [4, 5]. Metastasis is a multifaceted process involving the detachment of cancer cells from the primary tumor, migration, adhesion, and invasion via the basement membrane or extracellular matrix (ECM), surviving in the circulatory system, invasion into distant secondary organs or tissues, and subsequent proliferation [5]. The breakdown of the ECM facilitates tumor cell invasion, thereby promoting metastasis. Matrix metalloproteinases (MMPs) are a family of zinc-dependent endopeptidases that play a crucial role in cleaving basement membrane and ECM components, as well as in tumor cell invasion, metastasis, and angiogenesis [6]. MMP-2 (gelatinase A, 72 kDa) is an MMP family member that facilitates the process of metastasis. MMP-2 is implicated in ECM degradation and contributes to tumor cell growth, differentiation, invasion, metastasis, regulation of tumor angiogenesis, and immune surveillance. The upregulation of MMP-2 is observed in various tumors, and its elevation facilitates the proliferation, motility, and metastasis of malignant tumor cells [7, 8]. Therefore, the inhibition of MMP-2 is an effective strategy for preventing tumor cell metastasis [9].

Natural products and their derivatives are widely used in both conventional and modern drugs for the treatment of various diseases. Over 40% of new chemical substances arise from microorganisms [10]. Endophytic fungi are microorganisms that reside within the living tissues of plants, typically without causing any harmful or negative effects. Plants frequently host one or more endophytic fungus. The relationship between endophytic fungi and their hosts is mutually beneficial; hosts provide habitat and nutrients, whereas fungi produce functional metabolites that enhance plant growth and survival [11]. Endophytic fungi produce metabolites that exhibit structural diversity and a broad range of biological activities [12, 13]. The metabolites produced by endophytic fungi exhibit similarities to those generated by host plants, suggesting their potential as an alternative source of bioactive compounds [12]. Endophytic fungi have been produced many different types of secondary metabolites include alkaloids [14–16], phenolics [16, 17], flavonoids [17, 18], terpenoids [19], coumarins [20], lactones [21], and other polyketides [22]. These small molecules exhibit cytotoxic [23], antibacterial [24], antitrypanosomal [25, 26], enzyme (BACE1) inhibitory [27], anti-inflammatory activities [28, 29]. Previous studies have reported the anticancer activities of endophytic fungi extract on lung cancer cell. The ethyl acetate extracts from the endophytic fungus *H. fuscoatra* suppressed the growth, migration, and invasion of A549 lung cancer cells [30]. The mangrove-derived endophytic fungi significantly suppressed the growth and angiogenesis of lung cancer cell lines A549 and NCI-H460 [31]. The endophytic fungi extracts from mangrove plants *Rhizophora stylosa* and *R. mucronata* displayed cytotoxicity against A549 lung cancer cells [32]. Terrein from endophytic fungus *Aspergillus terreus* (JAS-2) exhibited anticancer activity against human lung cancer cell line A549 [33]. The genus Aspergillus is a common endophytic fungus present in various plants, including mosses, ferns, liverworts, and hornworts, spreading from tropical regions to the arctic tundra. It exhibits significant chemical diversity that includes alkaloids, sterols, diphenyl ethers, terpenoids, phenalenones, xanthones, cytochalasins, pyrones, and butenolides, which demonstrate a wide range of bioactivities, such as antibacterial, antifungal, anticancer, anti-inflammatory, and cytotoxic effects [34]. *Aspergillus aculeatus* is a member of the genus Aspergillus. It serves as a powerful producer of diverse bioactive substances. Previous studies demonstrated the secondary metabolites from *A. aculeatus* have anticancer activities on human epidermoid carcinoma in the mouth (KB), human breast cancer (MCF-7), and human lung cancer cells (NCI-H187) [35]. This study examined the effects of the extract from endophyte *A. aculeatus*, isolated from *C. annuum* L., on antiproliferation, antimigration, and anti-invasion in lung cancer. Subsequently, an *in silico* method was employed to predict the activity of specific proteins interacting with bioactive compounds. Molecular docking investigations were performed on secondary metabolite compounds to determine their potential to inhibit MMP-2.

## 2. Materials and Methods

### 2.1. Chemicals and Reagents

Powdered Dulbecco's modified Eagle's medium (DMEM), FBS, antibiotics solution, 1 × 0.25% trypsin was purchased from Gibco. Dimethyl sulfoxide (DMSO), 3-(4,5-dimethylthiazol-2-yl)-2,5-diphenyl-2H-tetrazolium bromide (MTT), and gelatin A were purchased from Sigma-Aldrich (St. Louis, MO, USA). Phosphate-buffered saline was purchased from Merck Millipore Corp (Darmstadt, Germany). Matrigel matrix was purchased from Corning incorporated (Bedford, MA, USA).

### 2.2. Sample Collection and Fungal Isolation

The plant sample *Capsicum annuum* L. was collected from the botanical garden of Walailak University in 2022. The specimen was identified at the Walailak Herbarium with voucher number 01569. Healthy leaves were collected randomly, placed into zip plastic bags, stored with ice, and transported to the laboratory. Leaves were rinsed with running tap water to eliminate adhered epiphytes. Following drying under sterile conditions, surfaces were disinfected through sequential immersion in 70% ethanol for 5 min. Leaves were randomly cut into 0.5-cm pieces (10 pieces per leaf) using a hole puncher under aseptic conditions. The sections underwent immersion in 95% ethanol for 1 min, followed by exposure to a 3% sodium hypochlorite solution for 3 min, and finally immersed in 95% ethanol for 0.5 min. All samples underwent rinsing with sterile distilled water and were subsequently dried on sterile paper. Leaf sections were plated in the SDA medium pH = 5.6 containing chloramphenicol and incubated at 25 ± 2°C for 1–3 days. Upon the appearance of hyphal tips in any fungal colony during incubation, the colony was transferred to new SDA plates for further incubation to obtain pure cultures.

### 2.3. Morphology Identification of Endophytic Fungi

Fungal isolates were identified morphologically according to previous studies [36]. Briefly, the slides were prepared from cultures and stained with lactophenol cotton blue reagent and examined with a bright-field microscope. Morphological identification was conducted based on growth patterns, hyphae characteristics, colony and medium coloration, surface texture, margin features, aerial mycelium presence, sporulation, acervuli production, medium coloration, and conidia size and coloration, utilizing standard identification manuals [37].

### 2.4. Molecular Identification of Endophytic Fungi

Endophytes were characterized using the molecular identification technique using the internal transcribed spacer (ITS) gene according to the molecular biological protocol [38, 39]. Briefly, fungal hyphae measuring 0.5–1.0 cm^2^ were harvested from the Petri dish and subsequently lyophilized in a 2-mL tube (Eppendorf, Germany). The lyophilized fungal mycelia were ground, and the infection was interrupted. Fungus DNA was isolated following the manufacturer's protocol utilizing the fungus genomic DNA Kit (Geneaid, Taiwan). The isolated DNA was subsequently amplified using polymerase chain reaction (PCR). The primers ITS5 5′(GGA AGT AAA AGT CGT AACAAG G) 3′ and ITS4 5′(TCC TCC GCT TAT TGA TAT GC) 3′ were utilized for the PCR. The PCR used 20 ng of genomic DNA as the template in a 30-μL reaction mixture, employing EF-Taq (SolGent, Korea) with the following protocol: activation of Taq polymerase at 95°C for 5 min, followed by 31 cycles of 95°C for 0.5 min, 57°C for 0.5 min, and 72°C for 1.4 min, finishing with a 10-min extension at 72°C. The amplification products were purified using a multiscreen filter plate (Millipore Corp, USA). A sequencing reaction was conducted with a PRISM BigDye Terminator v3.1 Cycle Sequencing Kit. The DNA samples with the extension products were incorporated into Hi-Di formamide (Applied Biosystems, USA). The mixture was incubated at 95°C for 5 min, thereafter placed on ice for 5 min, and then analyzed using the ABI Prism 3730XL DNA analyzer (Applied Biosystems, USA).

### 2.5. Endophytic Fungal Cultivation and Extraction

Endophytic isolate was cultured on SDA at a temperature of 25 ± 2°C for 7 days. Four mycelial agar plugs (0.5 cm × 0.5 cm) were inoculated into two distinct culture flasks, each containing 175 mL of Sabouraud dextrose broth (SDB). The culture was shaken at 200 rpm at 25 ± 2°C for 14 days. After incubation, the cultures were filtered using Whatman No. 1 filter paper. The culture was evaporated using a rotary vacuum evaporator, followed by drying at 45°C for 24 h. The evaporated extracts were subsequently dried using freeze-drying (FDU-2100; Eyela, Tokyo, Japan) at −80°C for 36 h [40]. The dried material was subsequently powdered, weighed, and packaged in zip lock bags and then stored at 4°C for further study.

### 2.6. Chemical Compound Analysis via Liquid Chromatography–Mass Spectrometry (LC–MS)

To identify bioactive compounds in *A. aculeatus* extract, LC–MS was employed at the Medicinal Plants Innovation Center of Mae Fah Luang University, Thailand. The characterization of bioactive compounds by LC–MS was performed by an expert of the Scientific and Technological Instruments Center, Mae Fah Luang University. An Agilent 1290 series HPLC (Agilent Technologies, CA, USA) performed with an Agilent 6540 Accurate-Mass Q-TOF LC/MS (Agilent Technologies, CA, USA) equipped with an ESI source; a diode-array detector (DAD) was used in this work. The separation was achieved by an Agilent Poroshell 120 EC-C18 (4.6 × 150 mm, 2.7 μm) column, maintained at 35°C and with a flow rate of 200 μL/min. The column temperature was maintained at 35°C with a flow rate of 200 μL/min, and the injection volume was 1 μL. MS/MS analyses were carried out in the automatic mode with collision energy (10, 15, and 30 eV) for fragmentation. Peak identification was performed in both positive and negative modes, while the instrument control, data acquisition, and processing were performed using the Agilent Mass Hunter workstation software package (Qualitative Analysis, Version 8.08.00, Agilent) and Personal Compound Database and Library (PCDL). This study uses the method of Tedasen et al. and Phosri et al., and the method description partly reproduces their wording [41, 42].

### 2.7. Target Prediction

The potential targets for the studied compounds in humans were identified using SwissTargetPrediction, an online tool dedicated to predicting targets for bioactive small molecules [43]. This method is based on the principle that bioactive molecules with structural or functional similarities are likely to bind to analogous targets [44, 45]. By leveraging this concept, the approach predicts possible targets for a given molecule by associating it with proteins that have known ligands sharing significant similarity to the compound in question.

### 2.8. Ligand Structure Preparation

The three-dimensional structures of the ligands were obtained from the PubChem database [46]. Subsequent preparation involved adding polar hydrogens, assigning Gasteiger charges, and merging nonpolar hydrogens [47]. After preparation, the ligands were saved in PDBQT format. These preparatory steps were carried out using AutoDock Tools (ADT) Version 4.2 [48, 49].

### 2.9. Preparation of Human Matrix Metalloproteinase-2 (MMP-2) Structure

The crystal structure of human MMP-2, identified by PDB ID 8H78, was obtained from the Protein Data Bank (https://www.rcsb.org/), with a resolution of 2.40 Å derived via X-ray diffraction. For docking preparations, the protein structure was dehydrated to expose its amino acid residues, followed by the removal of all cocrystallized ligands to clear the binding site for a new ligand. Subsequently, polar hydrogens and Kollman charges were added, and nonpolar hydrogens were merged [50]. The prepared ligands were then saved in PDBQT format. These modifications were conducted using ADT Version 4.2 [48, 49] and further supported by using the BIOVIA Discovery Studio Visualizer [51].

### 2.10. Molecular Docking Simulations

The docking procedure utilized AutoGrid4 to create precise grid maps of the active site [48]. Grid parameters were set with a spacing of 0.375 Å, and the grid box was centered at coordinates *x*: −7.59, *y*: 11.25, and *z*: 1.24, enclosing the protein within a 60 × 80 × 60 Å grid along the *x*, *y*, and *z* axes. Genetic algorithm (GA) parameters were carefully optimized, including a population limit of 200, a maximum of 2.5 million energy evaluations, a mutation rate of 0.02, a crossover rate of 0.80, and an elitism value of 1.0. Each docking simulation involved 50 runs using the GA [52, 53]. To enhance docking precision, a combined approach was adopted, employing both local searches (Solis and Wets method) and global searches (GA) through the Lamarckian GA [54]. Each ligand underwent 10,000 independent docking trials, repeated five times for greater accuracy. ADT Version 4 was used to calculate the protein-ligand binding energy (ΔGbind) and the inhibitory constant [48, 49].

### 2.11. Protein and Ligand Visualizations

The Mol^∗^ Viewer tool [55] was employed for visualizing proteins and ligands, providing a comprehensive and detailed representation of molecular structures. This visualization facilitated valuable insights into three-dimensional orientations and intermolecular interactions.

### 2.12. Cell Culture

The A549 cells were obtained from the American Type Culture Collection (ATCC, Manassas, VA) and cultured in DMEM containing 10% FBS, 100 U/mL penicillin, and 100 μg/mL streptomycin at 37°C in a humidified atmosphere with 5% CO_2_. The study was approved by the Institutional Biosafety Committee of the Faculty of Science at the Prince of Songkla University, Thailand.

### 2.13. Cell Viability Determination

The MTT assay was employed to examine the impact of *A. aculeatus* extract on cell viability. A549 cells were cultured at a density of 7 × 10^3^ cells/well in a 96-well plate for 24 h. The cells were subsequently exposed to different concentrations of extract (0–1000 μg/mL) for 24 h. Following incubation, 0.5 mg/mL MTT solution was added to each well, and the plates were incubated for 2 h at 37°C. To solubilize water-insoluble purple formazan crystals, 100 μL of DMSO was added to each well. Absorbance was measured with a microplate spectrophotometer at 570 nm, and the survival percentage was calculated relative to the control [56].

### 2.14. Wound Healing Assay

A549 cells were plated on 12-well plates at a density of 2 × 10^5^ cells using the culture medium and cultured for 24 h. After the cells reached confluence, a 200-μL tip was used to scrape through the center of the well to form a wound. Cells were rinsed twice with the culture medium to remove any suspended cell debris. Monolayer cells were incubated with *A. aculeatus* extract at nontoxic concentrations (0, 15.63, 31.25, and 62.5 μg/mL) at 37°C for 24 h. Following incubation, wound closure was assessed, and images were obtained using a phase-contrast inverted microscope. The wound area was assessed using ImageJ software (NIH, Bethesda, MD) [4].

### 2.15. Cell Migration and Invasion Assay

Cell migration and invasion were performed using transwell chambers with polyethylene terephthalate (PET) filters containing an 8-μm pore size (Corning Costar, Cambridge, MA, USA) in 24-well plates. In order to perform the cell migration assay, cells were seeded in the upper chamber at a density of 3 × 10^4^ cells/well in serum-free media after being pretreated with 15.63, 31.25, and 62.5 μg/mL of *A. aculeatus* extract and 0.5% DMSO as control. DMEM with 10% FBS was added to the lower transwell chamber to act as the chemoattractant. After 24 h of incubation, cells on the upper surface of the filter were removed with a cotton swab. Then, cells on the lower surface of the filter were fixed with methanol and stained with 0.5% crystal violet. Migratory cells were observed using an inverted microscope, and five randomly selected points from each sample were chosen. The percentage of migratory cells for each treatment was measured using ImageJ software relative to the control group [56].

The transwell chamber for the invasion assay was precoated with 30 μg of Matrigel (Corning Incorporated, Bedford, MA, USA) overnight at 37°C. The invasion procedure was similar to the migration procedure.

### 2.16. Cell Adhesion Assay

Cells were pretreated with various doses of *A. aculeatus* extract at nontoxic concentrations (15.63, 31.25, and 62.5 μg/mL) along with 0.5% DMSO as a control for 24 h, and after that, 1 × 10^4^ cells/well were placed into a Matrigel-coated 96-well plate for 1 h. Following the incubation time, the nonadherent cells were eliminated using PBS. The adhering cells were incubated with 0.5 mg/mL of MTT solution at 37°C for 3 h. Subsequently, 100 μL of DMSO was applied to each well to solubilize the water-insoluble purple formazan crystals. Absorbance was measured at 570 nm with a microplate spectrophotometer. The percentage of cell adhesion was determined in relation to the control [56].

### 2.17. Gelatin Zymography

Gelatin zymography was performed for determining MMP-2 activity in culture media. The experimental procedure was carried out as previously described by Tajhya et al. Briefly, the conditioned media were mixed with loading buffer and analyzed using 7.5% SDS-PAGE containing 0.1% gelatin. The gels were electrophoresed, rinsed with 2.5% Triton X-100, and then incubated for 48 h at 37°C in zymogram incubation buffer (50 mM Tris-HCl, pH 7.6, 10 mM CaCl_2_, 50 mM NaCl, 0.05% Brij 35). The gel was subsequently dyed with Coomassie brilliant blue R-250. The bands of gelatinolytic activity were measured utilizing NIH ImageJ program [57].

### 2.18. Statistical Analysis

Data were reported as the mean ± standard deviation (SD) of three independent experiments. Statistical significance was analyzed by one-way ANOVA test and Student's *t*-test. The differences between the treatment groups and the untreated group were considered statistically significant at *p* < 0.05.

## 3. Results

### 3.1. Isolation and Identification of Endophytic Fungus

**Figure 1:**
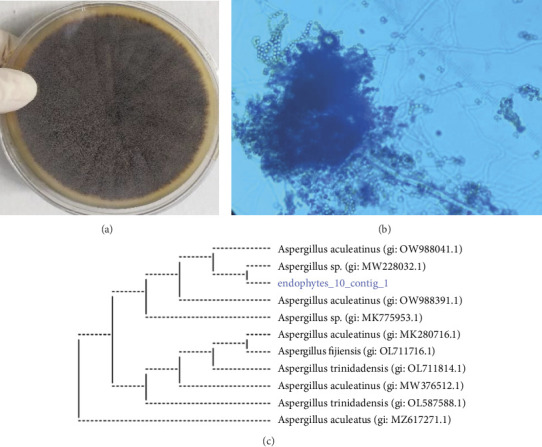
Morphology of the endophytic fungi isolate. (a) Colonies on Sabouraud dextrose agar. (b) The microscopic feature of the isolated endophytic fungus was stained with lactophenol cotton blue. (c) Molecular identification of endophytes was performed by sequencing ITS 4 and 5 regions of *Aspergillus aculeatus*.

Morphological analysis and microscopic observations identified the endophyte 10 as *Aspergillus* spp. (Figures 1(a) and 1(b)). Molecular identification of endophytes was conducted through sequencing the ITS 4 and 5 regions of the fungal genome. The ITS fragment, measuring approximately 570 bp, was amplified and sequenced. NCBI BLAST analysis indicated that the query sequence exhibited a high degree of homology with *Aspergillus aculeatus* (accession number: KJ958359.1), demonstrating 99% sequence similarity. A phylogenetic tree was generated utilizing the endophyte ITS region (Figure 1(c)).

### 3.2. Chemical Profiling of Endophytic Fungus *A. aculeatus* Isolated From *C. annuum* L. by LC–MS

**Figure 2:**
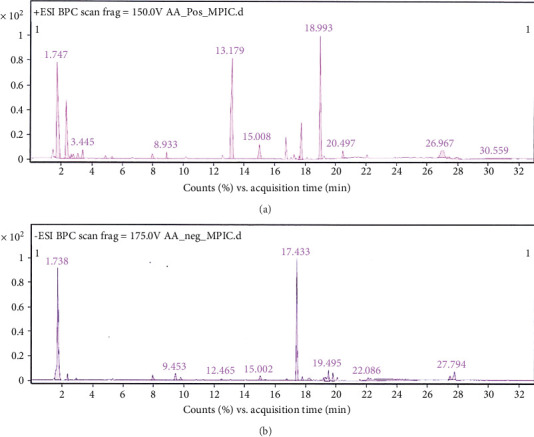
LC–MS chromatogram of compounds of *A. aculeatus* extract isolated from *C. annuum* L. using the positive ionization mode (a) and the negative ionization mode (b).

LC–MS represents a contemporary and effective method for the identification of compounds in biological samples [58]. This study evaluated the extract of the endophytic fungi *A. aculeatus* isolated from *C. annuum* L. using the LC–MS technique. The retention time and mass spectra of the extract fractions were analyzed in comparison to mass spectra from the data library ScienceDirect, SciFinder, and Scholar. The positive ionization mode identified 22 bioactive compounds, while the negative ionization mode identified 32 bioactive compounds in the *A*. *aculeatus* extract that was separated from *C. annuum* L. The list of substances that were evaluated using LC–MS is shown in Tables 1 and 2, which are backed up by Figures 2(a) and 2(b), respectively.

### 3.3. Target Prediction

SwissTargetPrediction is a widely utilized online platform for the identification of biological targets associated with bioactive small molecules in humans and other vertebrate species. In the present study, compounds derived from endophytic fungi were subjected to analysis to identify potential interactions with human MMP-2. The findings revealed that five compounds detected in the LC–MS chromatogram of *A. aculeatus* extract isolated from *C. annuum* L., under the positive ionization mode, were predicted to target MMP-2. These compounds included asperfuran, 2-hydroxy-7-methyl-2-propan-2-ylfuro[3,2-h] isoquinolin-3-one, flavoglaucin, tajixanthone methanoate, and gartryprostatin C. Similarly, five additional compounds identified under the negative ionization mode, namely, flavoglaucin, Cyclo-(Ala-Val), N-acetyltyramine, tryprostatin A, and aspernigerin, were also predicted as potential ligands for human MMP-2 (Supporting Files 1 and 2). Consequently, these compounds were further analyzed for their binding affinities and interaction profiles through molecular docking simulations.

### 3.4. Molecular Docking Simulations

**Figure 3:**
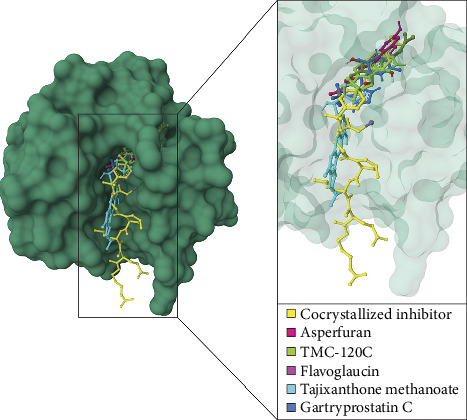
A structural depiction illustrates the binding interactions between human MMP-2 and various ligands, including a cocrystallized inhibitor (yellow) and natural compounds identified from endophytic fungi using LC–MS chromatography in the positive ionization mode. The natural compounds include asperfuran (rose), TMC-120C (chartreuse), flavoglaucin (magenta), tajixanthone methanoate (cyan), and gartryprostatin C (azure). A magnified view of the active site is presented, emphasizing the spatial arrangement of the docked ligands in relation to the cocrystallized inhibitor.

**Figure 4:**
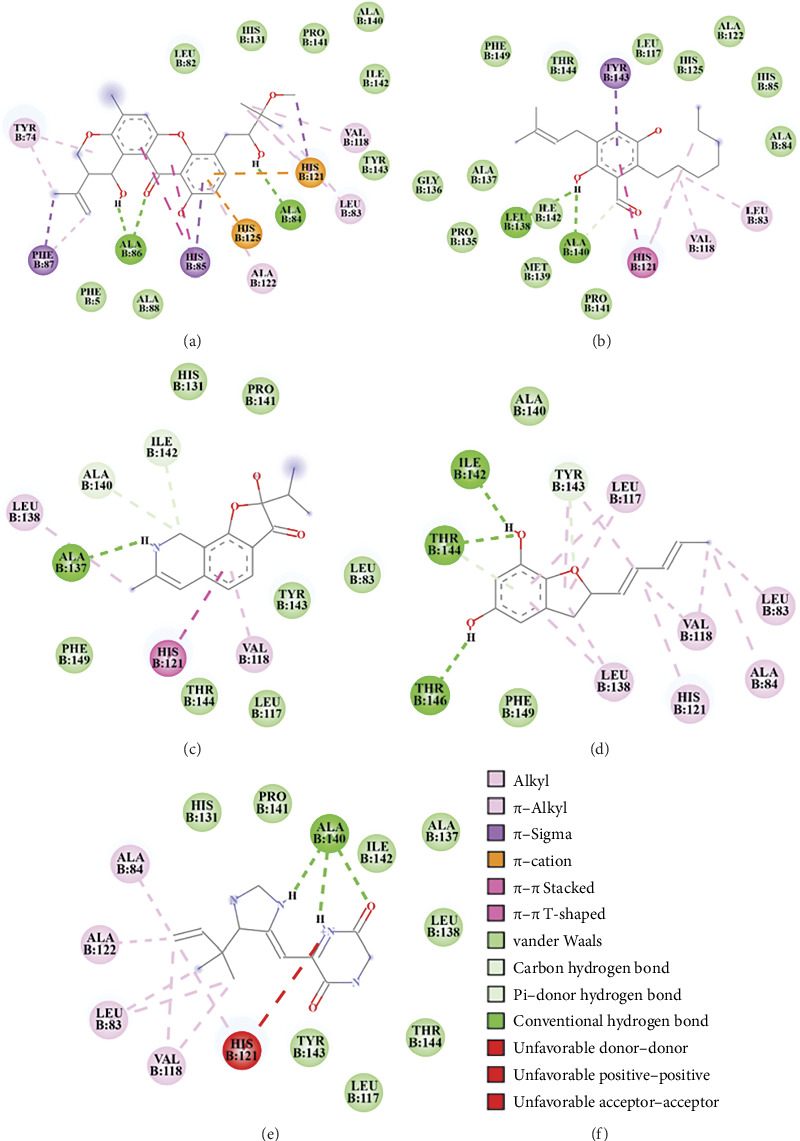
Two-dimensional interaction diagrams illustrating the binding modes of natural compounds identified from endophytic fungi through LC–MS chromatography in the positive ionization mode, within the active site of human MMP-2. Panels A to E correspond to specific ligands: tajixanthone methanoate (a), flavoglaucin (b), TMC 120C (c), asperfuran (d), and gartryprostatin C (e). The various types of interactions are represented by different colors, as indicated in the legend (f).

Molecular docking simulations were performed on ten compounds to evaluate their interactions with human MMP-2 using AutoDock 4. Among the compounds detected in the LC–MS chromatogram of *A. aculeatus* extract isolated from *C. annuum* L. under the positive ionization mode, tajixanthone methanoate displayed the strongest binding affinity, followed by flavoglaucin, 2-hydroxy-7-methyl-2-propan-2-ylfuro[3,2-h]isoquinolin-3-one, asperfuran, and gartryprostatin C (Table 3; Figures 3 and 4).

Tajixanthone methanoate exhibited a binding energy of −8.80 kcal/mol and an inhibitory constant (Ki) of 357.21 nM. Interaction analysis revealed hydrogen bonds with Ala84 and Ala86, π–cation interactions with His121 and His125, π–π t-shaped interactions with His85, π–sigma interactions with His85, Phe87, and His121, alkyl or π–alkyl interactions with Tyr74, Leu83, Phe87, Val118, and Ala122, as well as van der Waals interactions with residues such as Ala140, Pro141, Ile142, Tyr143, Phe5, Leu82, Ala88, and His131 (Figure 4(a)). However, flavoglaucin demonstrated a binding energy of −8.43 kcal/mol with a Ki of 658.42 nM. It formed hydrogen bonds with Ala140 and Leu138, π–π stacked interactions with His121, π–sigma interactions with Tyr143, alkyl, or π–alkyl interactions with Leu83, Val118, and His121, and additional van der Waals interactions with residues including Ala84, His85, Leu117, Ala122, His125, Pro135, Gly136, Ala137, Met139, Pro141, Ile142, Thr144, and Phe149 (Figure 4(b)). The compound 2-Hydroxy-7-methyl-2-propan-2-ylfuro[3,2-h]isoquinolin-3-one (TMC 120C) exhibited a binding energy of −8.41 kcal/mol and a Ki of 689.75 nM. Key interactions included hydrogen bonds with Ala137, Ala140, and Ile142, π–π stacking with His121, alkyl, or π–alkyl with Val118 and Leu138 and van der Waals interactions Leu83, Leu117, His131, Pro141, Tyr143, Thr144, and Phe149 (Figure 4(c)). Asperfuran showed a binding energy of −8.25 kcal/mol and a Ki of 890.02 nM. Its interactions involved hydrogen bonding with Ile142, Tyr143, Thr144, and Thr146, alkyl, or π–alkyl interactions with Leu83, Ala84, Leu117, Val118, His121, and Leu138, as well as van der Waals interactions with Ala140 and Phe149 (Figure 4(d)) and Gartryprostatin C, with a binding energy of −5.64 kcal/mol and a Ki of 1.18 μM, exhibited hydrogen bonds with Ala140, alkyl, or π–alkyl interactions with Leu83, Ala84, Val118, His 121, and Ala122, unfavorable positive–positive interaction with His121, and van der Waals interactions with residues including Leu117, His131, Ala137, Leu138, Pro141, Ile142, Tyr143, and Thr144 (Figure 4(e)).

**Figure 5:**
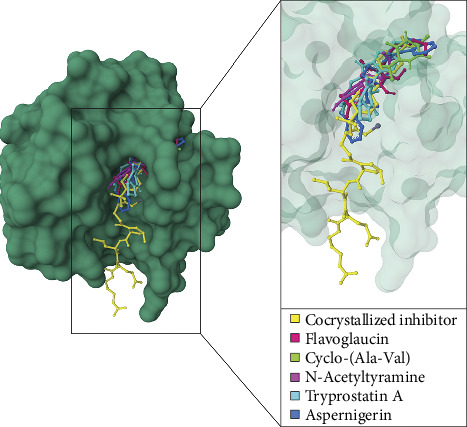
A structural illustration depicts the binding interactions of human MMP-2 with a cocrystallized inhibitor (yellow) and several natural compounds isolated from endophytic fungi and identified via LC–MS chromatography in the negative ionization mode. These natural compounds include flavoglaucin (rose), cyclo-(ala-val) (chartreuse), N-acetyltyramine (magenta), tryprostatin A (cyan), and aspernigerin (azure). An enlarged view focuses on the active site of human MMP-2, highlighting the spatial arrangement of the docked ligands in relation to the cocrystallized inhibitor.

**Figure 6:**
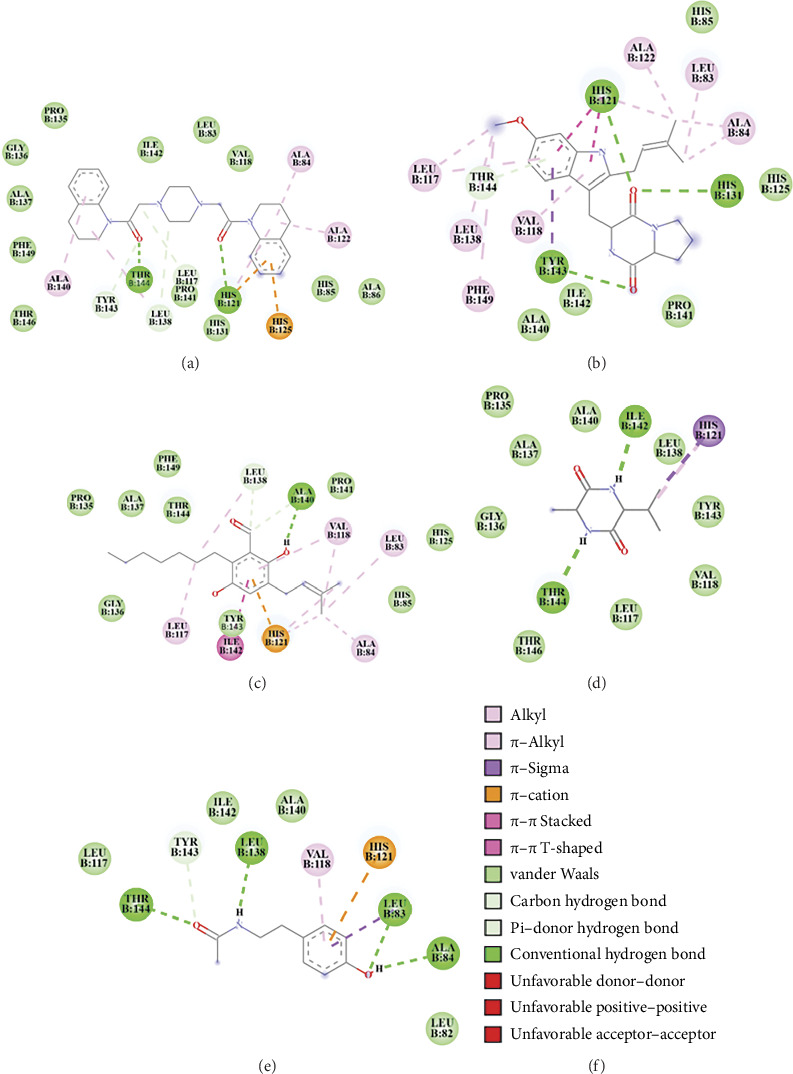
Two-dimensional interaction diagrams depict the binding modes of natural compounds isolated from endophytic fungi and identified through LC–MS chromatography in the negative ionization mode within the active site of human MMP-2. Panels (a) to (e) correspond to specific ligands: aspernigerin (a), tryprostatin A (b), flavoglaucin (c), cyclo-(Ala-Val) (d), and N-acetyltyramine (e). The types of interactions are denoted by different colors, as detailed in the legend (f).

For compounds detected in the LC–MS chromatogram under the negative ionization mode, aspernigerin displayed the highest binding affinity, followed by tryprostatin A, flavoglaucin, cyclo-(Ala-Val), and N-acetyltyramine (Table 4; Figures 5 and 6). Aspernigerin exhibited a binding energy of −12.95 kcal/mol and a Ki of 321.08 pM. Key interactions included hydrogen bonds with Leu117, His121, Leu138, Tyr143, and Thr144, π–cation interactions with His125, alkyl, or π–alkyl interactions with Ala84, Ala122, and Ala140, and van der Waals interactions with residues such as Leu83, His85, Ala86, Val118, His131, Pro135, Gly136, Ala137, Pro141, Ile142, Thr146, and Phe149 (Figure 6(a)). Tryprostatin A, with a binding energy of −10.78 kcal/mol and a Ki of 12.59 nM, formed hydrogen bonds with His121, His131, Tyr143, and Thr144, π–π stacked interaction with His121, π–sigma interaction with Tyr143, alkyl, or π–alkyl interactions with Leu83, Ala84, Leu117, Val118, Ala122, Leu138, and Phe149, and additional van der Waals interactions with His85, His125, Pro141, Ala140, and Ile142 as shown in Figure 6(b). However, flavoglaucin displayed a binding energy of −8.71 kcal/mol and a Ki of 410.87 nM. Its interactions included hydrogen bonding with Leu138 and Ala140, π–cation bonding with His121, π–π stacked or amide–π stacked interaction with Ile142, alkyl, or π–alkyl interactions with Leu83, Ala84, Leu117, Val118, His121, and Leu138, and van der Waals interactions with His85, His125, Pro135, Gly136, Ala137, Pro141, Tyr143, Thr144, and Phe149 (Figure 6(c)). Cyclo-(Ala-Val) showed a binding energy of −6.61 kcal/mol and a Ki of 14.4 μM, with hydrogen bonds involving Ile142 and Thr144, π–sigma and π–alkyl interactions with His121, and van der Waals interactions with residues Leu117, Val118, Pro135, Gly136, Ala137, Leu138, Ala140, Ile142, Tyr143, and Thr146 (Figure 6(d)), and N-acetyltyramine demonstrated a binding energy of −6.58 kcal/mol and a Ki of 15.08 μM. It formed hydrogen bonds with Leu83, Ala84, Leu138, Tyr143, and Thr144, π–sigma interaction with Leu83, π–cation interaction with His121, π–alkyl interaction with Val118, and van der Waals interactions with Leu82, Leu117, Ala140, and Ile142 (Figure 6(e)).

### 3.5. Effect of *A. aculeatus* Extract on A549 Cell Viability

**Figure 7:**
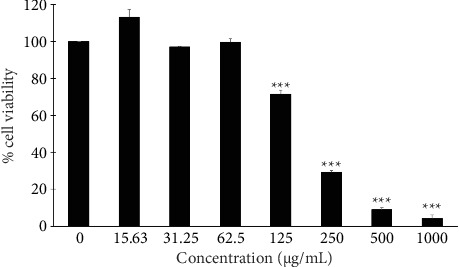
Effect of *A. aculeatus* extract on the viability of A549 cells. Cells were exposed to different concentrations of the extract for 24 h. The nontoxic concentrations of the extract were chosen for the subsequent experiments. Data are mean values ± SD of three independent experiments (*n* = 3). ^∗∗∗^*p* < 0.001 compared to the control group.

We assessed the cytotoxic effect of *A. aculeatus* extract on A549 cells by treating the cells with varying concentrations of the extract for 24 h. The extract demonstrated a dose-dependent reduction in A549 cell viability, with an IC_50_ of 286.36 ± 122.57 μg/mL (Figure 7). The extract at concentrations between 15.63 and 62.5 μg/mL did not influence the viability of A549 cells. Consequently, concentrations of 15.63, 31.25, and 62.5 μg/mL of *A. aculeatus* extract were selected for subsequent studies.

### 3.6. Effect of *A. aculeatus* Extract on the Suppression of Cell Migration in A549 Cells

**Figure 8:**
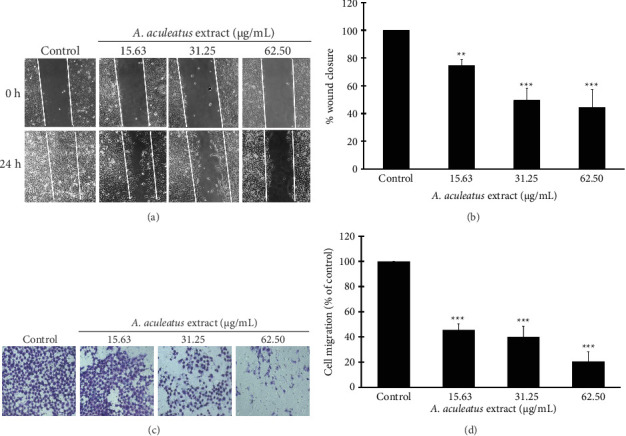
*A. aculeatus* extract inhibited the A549 cell migration as determined by wound healing and transwell chamber assay. (a) The wound area of A549 cells was measured at 0 and 24 h following treatment with *A. aculeatus* extract 15.63, 31.25, and 62.5 μg/mL, and 0.5% DMSO as control. (b) The wound closure was measured by measuring the area between the edges. (c) Transwell chamber analysis showed that *A. aculeatus* extract dose-dependently inhibited cell migration. (d) The migratory cell was calculated by NIH ImageJ. Data are shown as the mean ± SD of three independent experiments (*n* = 3). ^∗∗^*p* < 0.01, ^∗∗∗^*p* < 0.001 vs. control group.

Cell migration was assessed with the wound-healing and the transwell migration assay. Cell motility towards the wound area was markedly diminished after 24 h of treatment with 15.63, 31.25, and 62.5 μg/mL of *A. aculeatus* extract, as shown in Figures 8(a) and 8(b). The transwell migration assay revealed that *A. aculeatus* extract markedly suppressed cell migration in a dose-dependent fashion (Figures 8(c) and 8(d)). The results indicated that *A. aculeatus* extract markedly impeded cell migration, as shown by diminished wound closure and decreased cell migration in A549 treated cells.

### 3.7. Effect of *A. aculeatus* Extract on Cell Invasion Ability in A549 Cells

**Figure 9:**
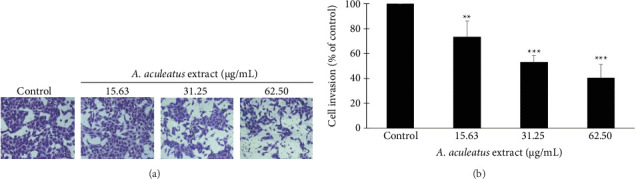
Effect of *A. aculeatus* extract on cell invasion in A549 cells. (a) A549 cells were treated with 0.5% DMSO and 15.63, 31.25, and 62.5 μg/mL of *A. aculeatus* extract in a transwell chamber coated with Matrigel. The invasive cells were stained with 0.5% crystal violet and photographed under a microscope. (b) Percentage (%) of cell invasion was calculated in the treated cells compared to the untreated cells. The data represent mean results ± standard deviation from three different studies (*n* = 3). ^∗∗^*p* < 0.01, ^∗∗∗^*p* < 0.001 in comparison to the control group.

To investigate the effect of *A. aculeatus* extract on cell invasion, a transwell chamber coated with Matrigel assay was used. Matrigel is a gelatinous ECM protein mixture, commonly used in tumor therapeutic studies. The result in Figure 9 showed that *A. aculeatus* extract significantly inhibits the ability of A549 cells to pass through a Matrigel insert membrane. This result suggested that *A. aculeatus* extract suppressed cell invasion in lung cancer A549 cells.

### 3.8. Effect of *A. aculeatus* Extract on Cell Adhesion in A549 Cells

**Figure 10:**
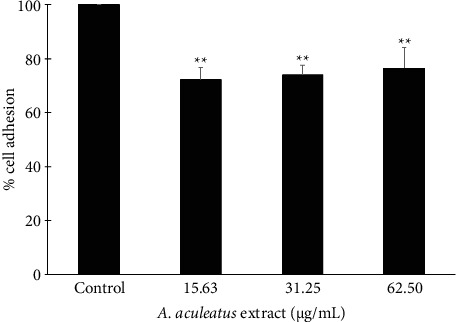
*A. aculeatus* extract markedly diminished the adhesive capacity of A549 cells to Matrigel in a dose-dependent fashion relative to the untreated control cells. The percentage of cell adhesion was calculated for treated cells in comparison to untreated cells. The data represent mean values ± SD from three independent experiments (*n* = 3). ^∗∗^*p* < 0.01 in comparison to the control group.

The effect of *A. aculeatus* extract on cell adhesion was assessed using a Matrigel-coated plate. Matrigel comprises a protein combination, including collagen, laminin, and fibronectin, which are widely present in the ECM. The findings indicated that 15.63, 31.25, and 62.5 μg/mL of *A. aculeatus* extract significantly diminished the adhesive capacity of A549 cells to Matrigel in a dose-dependent fashion relative to the untreated control cells, as shown in Figure 10. The extract of *A. aculeatus* at concentrations of 15.63, 31.25, and 62.5 μg/mL decreased cell adhesion by 72.23 ± 4.46%, 73.97 ± 3.77%, and 76.55 ± 7.74%, respectively, in comparison to the control group (*p* < 0.05). The results suggested that *A. aculeatus* extract could diminish cell adhesion in A549 cells.

### 3.9. Effect of *A. aculeatus* Extract on MMP-2 Activity

**Figure 11:**
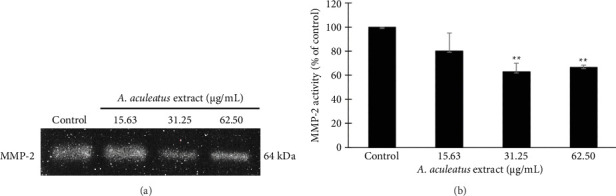
Effect of *A. aculeatus* extract on MMP-2 activity in A549 cells. (a) MMP-2 activity was assessed using gelatin zymography. (b) MMP-2 activity significantly decreased following treatment with 31.25 and 62.5 μg/mL *A. aculeatus* extract for 24 h. The results are presented as the mean ± SD from three independent experiments. ^∗∗^*p* < 0.01 in comparison to the control group.

The activity of MMP-2 was assayed using gelatin zymography. As shown in Figure 11, MMP-2 activity was significantly reduced after treatment with 31.25 and 62.5 μg/mL *A. aculeatus* extract. This result indicated that MMP-2 activity of A549 lung cancer cells was dramatically reduced by *A. aculeatus* extract. Therefore, this result indicated that *A. aculeatus* extract suppressed cell migration and cell invasion via suppressing MMP-2 activity.

## 4. Discussion

Endophytic fungi are microorganisms that inhabit the living tissues of plants, generally without inducing any deleterious effects. For over 30 years, extracts from fungal endophytes associated with specific medicinal plants have been utilized for treatment of various diseases. The production of metabolites by fungal endophytes is particular to genus or species and can differ depending on abiotic environments and biotic factors such as infections. Many synthetic pharmaceuticals exist for cancer treatment. The side effects of synthetic pharmaceuticals, the drug-resistant characteristics of carcinoma cells, the absence of cancer cell-specific medications, and the limitations of chemotherapeutic agents present significant challenges in cancer treatment [11]. Fungal endophytes associated with plants represent a crucial and underutilized source of natural bioactive metabolites. Several fungal endophytes have demonstrated significant potential as sources of anticancer compounds [59–61]. Herein, we isolated an endophytic fungus from the leaves of *C. annuum* L. The fungus was identified as *A*. *aculeatus* by the PCR-based ITS region amplification method. *A. aculeatus* is a member of the genus *Aspergillus*. The genus *Aspergillus* is a common endophytic fungus present in various plants. It serves as a powerful producer of diverse bioactive substances. Previous studies demonstrated that the secondary metabolites from *A. aculeatus* have anticancer activities on human epidermoid carcinoma in the mouth (KB), human breast cancer (MCF-7), and human lung cancer cells (NCI-H187) [35]. A recent study demonstrated that secondary metabolites from *A. aculeatus* MBT-102 isolated from the leaves of the *Rosa damascena* induced apoptosis and inhibited MDA-MB-231 triple negative breast cancer cells [10].

Cancer metastasis is a main cause of cancer-related death in lung cancer patients. It is a multifaceted process involving the detachment of cancer cells from the primary tumor, migration, adhesion, and invasion via the basement membrane or ECM, surviving in the circulatory system, invasion into distant secondary organs or tissues, and subsequent proliferation [5]. Inhibition of any metastatic step is a target for inhibiting the progression of cancer metastases. In this work, we examine the antimigration and anti-invasion activities of the *A. aculeatus* extract on lung cancer cells. We found *A. aculeatus* inhibited the migratory, invasive, and adhesive capabilities of A549 cells in a dose-dependent manner. *A. aculeatus* showed significantly decreased A549 cell viability, with an IC_50_ value of 286.36 ± 122.57 μg/mL. The extract at concentrations ranging from 15.63 to 62.5 μg/mL did not affect the viability of A549 cells. Noncytotoxic or subcytotoxic concentrations (which allows at least 80% cell survival) were selected for further investigation. A previous study demonstrated the cytotoxic effects of secalonic acid derivative F-7, derived from *A. aculeatus* MBT 102 and *Rosa damascena*, against various cancer cell lines: PC-3, MCF-7, HT-29, SW620, MDA-MB-231, and FR-2 cells [10]. Cell migration and invasion are critical processes in the spread of cancer cells. The ability of cancer cells to migrate and invade other tissues finally lead to metastasis. Herein, we demonstrated that *A*. *aculeatus* extract inhibited the migratory and invasion capabilities of A549 cells in a dose-dependent manner. Consistent with the previous study indicated that a secalonic acid derivative extracted from the endophytic *A. aculeatus* MBT 102, derived from *Rosa damascena*, induced apoptosis in MDA-MB-231 cells and reduced cell migration [10]. Cytochalasans derived from the endophytic fungus *Phomopsis* sp. shj2 had an antimigratory activity, with IC_50_ values ranging from 1.01 to 10.42 μM [62]. Perinadine A, derived from the Egyptian olive tree endophytic fungus *P. citrinum*, demonstrated excellent migratory inhibition against MDA-MB-231 cells [63]. Fumigaclavine C, extracted from the marine-derived fungus *A. fumigatus*, demonstrated significant suppression of cell migration and invasion by downregulating the protein and gene expressions of MMP-2 and MMP-9 in MCF-7 cells [64]. MMPs are the key proteases implicated in tumor cell motility, dissemination, tissue invasion, and metastasis [6]. Previous research showed the upregulation of MMP-2 expression associated with cancer invasion in various tumors [7, 8]. Downregulating MMP-2 expression can suppress tumor cell proliferation, growth, and metastasis, while enhancing tumor cell death [65–67]. Our results showed *A. aculeatus* extract significantly inhibited the enzyme activity of MMP-2. These findings suggested that *A. aculeatus* extract decreased cell migration and invasion of A549 cells by suppressing MMP-2 activity. In the present study, compounds derived from *A. aculeatus* were subjected to analysis to identify potential interactions with human MMP-2. Molecular docking simulations were performed to predict which compounds were responsible for the antimigration and anti-invasion activities. This study demonstrated that 22 chemical components were identified under the positive ionization mode, whereas negative ionization mode found 32 bioactive compounds, as shown in Tables 1 and 2. Five compounds under the positive ionization mode included asperfuran, 2-hydroxy-7-methyl-2-propan-2-ylfuro[3,2-h]isoquinolin-3-one, flavoglaucin, tajixanthone methanoate, and gartryprostatin C were predicted as potential ligands for human MMP-2. Among these compounds, tajixanthone methanoate displayed the strongest binding affinity with a binding energy of −8.80 kcal/mol and an inhibitory constant (Ki) of 357.21 nM. For compounds detected in the LC–MS chromatogram under the negative ionization mode, five additional compounds, namely, flavoglaucin, Cyclo-(Ala-Val), N-acetyltyramine, tryprostatin A, and aspernigerin, were also predicted as potential ligands for human MMP-2 (Supporting Files 1 and 2). Among these compounds, aspernigerin displayed the highest binding affinity with a binding energy of −12.95 kcal/mol and a Ki of 321.08 pM.

## 5. Conclusion

In conclusion, in this study, a fungal endophyte that has been isolated from *Capsicum annuum* L was identified and molecularly characterized as *A. aculeatus* for the first time. Molecular docking simulations showed that 10 compounds under positive and negative ionization modes displayed the binding affinity to MMP-2. We showed that the *A. aculeatus* extract inhibited A549 cell migration, invasion, and adhesion by inhibiting MMP-2 activity. According to these findings, the extract of endophytic *A*. *aculeatus* is a novel therapeutic drug for reducing cancer spread. However, further investigation, including isolation, characterization of the specific active compounds, and in-depth mechanistic studies, as well as in vivo studies, should be needed to exploit their potential to develop as a chemotherapeutic agent.

## Figures and Tables

**Table 1:** The list of compounds identified by the LC–MS of *A. aculeatus* extract in the positive mode.

No.	RT (min)	Mass	*m*/*z* (expected)	Chemical formula	Mass error (ppm)	Identification
1	1.486	142.0269	160.0608	C_6_H_6_O_4_	1.87	5-Acetoxymethylfuran-3-carboxylic acid
2	1.747	431.1601	454.1496	C_22_H_22_NO_8_	4.88	Pseurotin A
3	4.229	363.1600	381.1952	C_21_H_21_N_3_O_8_	4.75	Spirotryprostatin B
4	5.144	130.0631	153.0508	C_6_H_10_O_3_	0.80	Mevalolactone
5	6.646	218.0950	236.1287	C_13_H_14_O_3_	1.47	Asperfuran
6	8.018	257.1040	258.1110	C_15_H_15_NO_3_	−4.79	TMC-120C
7	8.018	218.0950	236.1288	C_13_H_14_O_3_	3.24	Asperfuran
8	8.606	396.1197	414.1533	C_22_H_20_O_7_	−3.04	6,8-di-O-Methyllaverufin
9	8.998	286.0855	287.0898	C_16_H_14_O_5_	4.87	Flavoglaucin
10	9.455	610.1510	633.1397	C_27_H_30_O_16_	−3.90	Rutin
11	10.043	366.1463	367.1560	C_22_H_22_O_5_	−1.09	Asperteretal A
12	10.370	454.1978	472.2327	C_26_H_30_O_7_	−3.07	Tajixanthone methanonate
13	11.676	260.1271	283.1151	C_13_H_16_N_4_O_2_	−0.78	Gartryprostatin C
14	12.003	295.1180	318.1082	C_15_H_19_O_6_	−0.40	Aspvanicin A
15	12.264	495.2244	496.2345	C_28_H_33_NO_7_	−2.64	Cytochalasin E
16	13.898	507.2641	525.2982	C_30_H_37_NO_6_	3.88	Cytochalasin D
17	19.124	380.2199	381.2271	C_21_H_32_O_6_	0.06	SF002-96-1
18	20.301	420.1719	438.2060	C_29_H_24_O_3_	−1.53	Dihydroauroglaucin
19	21.019	410.3562	428.3891	C_29_H_46_O	3.27	(22E, 24R) Stigmasta-5,7,22-trien-3-β-ol,
20	21.346	396.3374	419.3251	C_28_H_44_O	−4.63	Esgosterol
21	21.999	274.2292	292.2640	C_19_H_30_O	−1.80	5α-Androstan-3-one
22	25.398	379.1904	397.2258	C_22_H_25_N_3_O_3_	2.15	Fumitremorgin C

**Table 2:** The list of compounds identified by the LC–MS of *A. aculeatus* extract in the negative mode.

No.	RT (min)	Mass	*m*/*z* (Expected)	Chemical formula	Mass error (ppm)	Identification
1	1.791	460.1395	505.1377	C_24_H_20_N_4_O_6_	2.57	Tryptoquivaline I
2	1.791	286.0848	331.0830	C_16_H_14_O_5_	2.36	Flavoglaucin
3	4.538	154.0265	153.0195	C_7_H_6_O_4_	−0.60	2,4-Dihydroxybenzoic acid
4	4.803	142.0267	201.0407	C_6_H_6_O_4_	0.85	5-Acetoxymethylfuran-3-carboxylic acid
5	5.067	246.1008	291.0989	C_13_H_14_N_2_O_3_	1.60	*N*-β-Acetyl tryptamine
6	6.230	214.1324	259.1302	C_10_H_18_N_2_O_3_	3.23	Cyclo(L-prolinyl-L-valive)
7	6.970	196.0732	195.0663	C_10_H_12_O_4_	−1.99	Atraric acid
8	7.815	341.1745	400.1880	C_19_H_23_N_3_O_3_	1.50	(±)-Janoxepin
9	8.132	168.0903	227.1041	C_8_H_12_N_2_O_2_	2.60	Cyclo-(Ala-Val)
10	8.766	130.0627	175.0614	C_6_H_10_O_3_	−1.96	Mevalolactone
11	10.563	248.0683	307.0822	C_13_H_12_O_5_	−0.84	Oryzae in A
12	10.563	262.0842	307.0825	C_14_H_14_O_5_	0.19	7-Methoxy-3-(2-oxopropy)-5-hydroxymethyl-isocoumarin
13	10.616	290.0794	349.0931	C_15_H_14_O_6_	1.25	Gefelin
14	12.888	179.0948	224.0933	C_10_H_13_NO_2_	0.78	*N*- Acetyltyramine
15	13.099	210.0537	209.0455	C_10_H_10_O_5_	4.05	(3R)-6,7,8-Trihydroxymellein
16	13.152	200.1046	199.0974	C_10_H_16_O_4_	−1.50	Pestalotiolactone C
17	16.693	184.1100	243.1239	C_10_H_16_O_3_	0.34	Pestalotiolactone D
18	17.169	381.2050	426.2031	C_22_H_27_N_3_O_3_	−0.54	Tyrprostatin A
19	17.327	208.0730	207.0656	C_11_H_12_O_4_	−2.85	Maltoryzine
20	17.380	493.2566	492.2498	C_28_H_35_N_3_O_5_	−2.23	Paraherquamide
21	18.173	290.1880	349.2019	C_18_H_26_O_3_	−0.73	Allahabadolactone A
22	18.490	428.1842	427.1773	C_24_H_28_O_7_	1.62	14-Deacetyl parasiticolide A
23	18.543	286.0834	345.0976	C_16_H_14_O_5_	−2.44	Flavoglaucin
24	18.649	210.0902	209.0830	C_11_H_14_O_4_	4.89	6-Isovaleryl-4-methoxy-pyran-2-one
25	18.860	290.1875	335.1855	C_18_H_26_O_3_	−2.53	Allahabadolactone A
26	18.860	366.1469	365.1397	C_22_H_22_O_5_	0.60	Asperteretal A
27	19.125	380.2198	379.2117	C_21_H_32_O_6_	−0.32	SF002-96-1
28	20.023	348.2295	347.2230	C_21_H_32_O_4_	−1.49	Salimyxin B
29	20.499	432.2528	491.2675	C_26_H_32_N_4_O_2_	0.72	Aspernigerin
30	21.028	250.1567	309.1711	C_15_H_22_O_3_	−0.97	Versicolactone B
31	22.086	356.1978	355.1904	C_22_H_28_O_4_	−2.83	11α-Hydroxycanrenone
32	22.403	284.2713	283.2641	C_18_H_32_O_2_	−0.88	Stearic acid

*Note:* m/z = mass-to-charge ratio.

Abbreviation: RT = retention time.

**Table 3:** Binding affinity and inhibitory constants of natural compounds derived from endophytic fungi detected via LC–MS chromatography (positive ionization mode) against human MMP-2.

Compound name	PubChem CID	MMP-2 (PDB ID: 8H78)	
		Binding affinity (kcal/mol)	Inhibitory constant (Ki)
Asperfuran	11969970	−8.25	890.02 nM
2-Hydroxy-7-methyl-2-propan-2-ylfuro[3,2-h]isoquinolin-3-one	10467646	−8.41	689.75 nM
Flavoglaucin	119037	−8.43	658.42 nM
Tajixanthone methanoate^∗^	46883486	−8.80	357.21 nM
Gartryprostatin C	142908295	−8.09	1.18 uM

^∗^The strongest binding affinity against human MMP-2.

**Table 4:** Binding affinity and inhibitory constants of natural compounds derived from endophytic fungi detected via LC–MS chromatography (negative ionization mode) against human MMP-2.

Compound name	PubChem CID	MMP-2 (PDB ID: 8H78)	
		Binding affinity (kcal/mol)	Inhibitory constant (Ki)
Flavoglaucin	119037	−8.71	410.87 nM
Cyclo-(Ala-Val)	139895	−6.61	14.4 uM
N-Acetyltyramine	121051	−6.58	15.08 uM
Tryprostatin A	9929833	−10.78	12.59 nM
Aspernigerin^∗^	11561081	−12.95	321.08 pM

^∗^The strongest binding affinity against human MMP-2.

## Data Availability

The data that support the findings of this study are available from the first author upon reasonable request.

## Supporting Information

Supporting Information 1 Supporting File 1: Target prediction of natural compounds derived from endophytic fungi detected via LC–MS chromatography (positive ionization mode).

Supporting Information 2 Supporting File 2: Target prediction of natural compounds derived from endophytic fungi detected via LC–MS chromatography (negative ionization mode).
